# The epidemiology of multimorbidity in France: Variations by gender, age and socioeconomic factors, and implications for surveillance and prevention

**DOI:** 10.1371/journal.pone.0265842

**Published:** 2022-04-06

**Authors:** Joël Coste, José M. Valderas, Laure Carcaillon-Bentata

**Affiliations:** 1 Public Health France, Saint-Maurice, France; 2 Department of Family Medicine, National University Health System, Yong Loo Lin School of Medicine, National University of Singapore, Singapore, Singapore; University of Botswana, BOTSWANA

## Abstract

**Background:**

Robust public health and health system response to the increasing burden of multimorbidity worldwide requires detailed epidemiological examination of its key sociodemographic and geographic determinants. We investigated the role of gender, age and socioeconomic and geographic factors on multimorbidity (i.e., having two or more conditions) in the adult population in France and examined implications for surveillance and prevention.

**Methods:**

We used data from two large nationwide representative surveys with cross-sectional and longitudinal health and socio-demographic indicators, conducted in France between 2008 and 2014. Morbidity counts and frequent dyads/triads of conditions independently impacting mortality, activity limitations, and perceived health were investigated with regard to differences in gender, age, socioeconomic (education, occupation and income) and geography (size of the urban unit and region).

**Results:**

The component conditions of multimorbidity varied with gender and age. Women experienced multimorbidity 23–31% more frequently and at a younger age (5–15 years earlier) than men. Multimorbidity increased with age while its associations with most health indicators weakened with it. Multimorbidity was strongly and independently associated with socioeconomic indicators, with a strong inverse dose-response relationship with education, but less consistently with geographic factors.

**Conclusions:**

Multimorbidity has diverse and variable components and impacts across gender and age. It is strongly associated with socioeconomic factors, notably educational level, for which causality appears likely. Consideration of this diversity and variability, its common occurrence in dyads and triads, and its impact on health outcomes according to age and gender may contribute to efficient surveillance and support the identification of prevention strategies targeting middle-aged men and women.

## Introduction

During the past decades, the extension of life expectancy and ageing of populations have increased the burden of chronic conditions, and multimorbidity (defined as having two or more conditions) has become a public health issue worldwide [1, 2]. As a result, it has been researched in various settings and populations. However, the wide range of approaches used to the measurement of multimorbidity has seriously compromised the ability to compare findings across time and countries [3, 4]. Furthermore, the impact of multimorbidity on relevant health outcomes such as mortality, activity limitations, and perceived health has rarely been investigated [5–7], and crucial research on determinants of multimorbidity such as gender, age and socioeconomic and geographic factors has not been consistently conducted [8, 9]. We recently proposed a new approach where multimorbidity assessment moves beyond counting conditions and pays special attention to the main multimorbid combinations (dyads or triads), their impact and joint effects on adverse health outcomes (mortality, activity limitations, and perceived health), and their etiological pathways which illuminate the *aggregation process* which drives multimorbidity. We suggested that multimorbid combinations with large health impacts, most deleterious interactions or shared risk factors [10] should be primarily considered. Using the same approach and nationwide representative surveys from France, the present study aimed to further assess the prevalence and component conditions of multimorbidity as well as the impact of multimorbidity on adverse outcomes according to gender and age and to examine relationships between multimorbidity and socioeconomic and geographic factors. These elements have not been previously investigated in France and only seldom in Europe. The purpose of this work was to draw more detailed implications for surveillance and prevention, specifically, which indicators would be more appropriate and which actions or targets (populations) should be prioritized according to their relevance.

## Materials and methods

### Survey designs, populations studied and collected data

Data from two large nationwide representative surveys recently conducted in France including similar lists of self-reported chronic conditions and health indicators were used: 1) The Disability Healthcare Household Survey from 2008 (Enquête Handicap–Santé Ménages, HSM), a purely cross-sectional two-stage survey conducted in 2008 with a focus on health, disability, and dependency; 2) the Health, Health Care, and Insurance Survey from 2010 and 2014 (Enquête Santé et Protection Sociale, ESPS), a longitudinal health survey representative of individuals living in households in France in 2010. Participation rates were high (80% and 77% for the two stages of HSM and 65% for ESPS), leading to 23,348 and 14,875 participants aged ≥ 25 years residing in France being included, respectively. Both surveys recorded similar lists of chronic or recurrent conditions (N = 61, ESPS survey considered conditions occurring in a 1-year period, whereas HSM survey considered lifetime occurrence), age (years) and gender (male, female), and socioeconomic indicators: education level (3 categories: less than secondary, secondary, and tertiary), employment grade (4 categories: manager or professional, middle manager or teacher, manual worker, no occupation or studying), income (3 tertiles if provided, otherwise “not provided”), size of the urban unit (9 categories), and geographic area (11 regions). The surveys also collected health status indicators, including the European Union Global Activity Limitation Indicator (GALI) and Self-Reported Health (SRH), and difficulties in activities of daily living/instrumental activities of daily living (ADL, N = 7, IADL, N = 12). Details on these surveys were previously reported [10].

### Morbidity assessment

Based on our previous findings [10], only the 48 (of 61) chronic conditions consistently associated with having an independent impact on health status indicators across surveys and/or within surveys across indicators were included in the multimorbidity analyses. In all analyses, four thresholds for the morbidity count were considered (number of conditions including and exceeding 1, 2, 3, 4). Multimorbidity was defined as a count ≥ 2. All dyads and triads of chronic conditions whose frequency was ≥ 0.50% were also considered.

### Outcomes

Health status indicators of interest in this study included GALI in 3 categories (severely limited/limited but not severely/not limited at all), SRH in 3 categories (very good or good/fair/bad or very bad), limitations in ADL and IADL in 3 categories (no limitation/Limitation in < 3 ADLs and < 2 IADLs/ Limitation in ≥ 3 ADLs and ≥ 2 IADLs), and mortality. Change scores for functioning and perceived health measures were calculated in ESPS subjects when repeated measurements were available (GALI, SRH). Half of the participants of ESPS (N = 7,727, one per household) were scheduled to be followed up and re-interviewed in 2014. Linkage with vital statistics allowed mortality (but not causes of death) to be assessed up to 2014 for these participants only.

### Statistical analysis

Due to the different timeframe used to investigate morbidities, no cross-validation could be carried out in the strictest sense. However, there were enough commonalities between the studies to seek convergent evidence from their parallel analyses, which were therefore systematically performed.

The four morbidity count thresholds and most frequent dyads and triads were considered with respect to their associations with age, gender, health status, socioeconomic and geographic indicators. Multiple binary (for dyads and triads) or polytomous (for morbidity count) logistic regression models were used to estimate odds ratios (OR) and 95% confidence intervals (95% CI) describing these associations, adjusting for relevant covariates in each analysis. Heterogeneity of effects and associations and non-linear trends were assessed using interaction terms and restricted cubic splines, respectively.

All the analyses were performed separately for the two surveys using SAS, version 9.2 software (SAS Institute Inc.). Appropriate weights were used to provide valid estimates for the French population (2008 for HSM, 2010 for ESPS), while taking into account the unequal probabilities of selection resulting from sample design, non-response, and non-coverage in both surveys [11, 12]. To produce robust results, stratified analyses by gender and age group (3 or 7 groups when possible) were mostly conducted in the largest sample from the HSM survey, as analysis implying dyads and triads of conditions.

### Ethics

This study was conducted following the guidelines set out in the Declaration of Helsinki. ESPS and HSM surveys were recognized to be of public health interest by the National Council for Statistical Information (CNIS), and their methodology was approved by the French Data Protection Authority (CNIL). All participants received an information letter before the start of the survey and provided written informed consent. The analyses presented here needed no further ethical approval.

## Results

The main characteristics of the two population samples are presented in Table 1. These samples were very similar as they were both assembled to reflect the general population in 2008–10.

**Table 1:** Description of the samples studied (ESPS and HSM surveys). All figures are weighted percentages unless otherwise indicated.

	HSM Survey sample (N = 23,348)	ESPS Survey sample (N = 14,875)
Sex, female	52.8	52.9
*Age*		
25–34 years	17.8	19.0
35–44 years	21.3	21.5
45–54 years	20.3	19.5
55–64 years	17.7	18.6
65–74 years	11.8	11.6
75–84 years	8.6	7.9
≥ 85 years	2.6	2.0
*Education*		
Less than secondary	30.7	33.3
Secondary	44.6	41.2
Tertiary	24.7	25.5
*Marital status*		
Married/living with a partner	71.7	70.9
Separated/divorced/Widowed	14.0	15.5
Single	14.3	13.6
*Employment status*		
Paid employment	55.1	58.7
Unemployed	5.2	6.0
Homemaker	6.6	5.5
Retired	29.2	27.4
Other	3.9	2.4
*Occupation (present or past)*		
Manager, professional	15.4	17.1
Middle manager, teacher	41.6	37.9
Manual worker	41.8	41.6
No occupration or studying	1.2	3.3
*Household incomes*		
Lower third	32.0	33.5
Middle third	34.5	32.6
Upper third	33.5	33.9
Missing (Number of)†	1,895	4,718
*Size of the urban unit*		
Less than 2,000 inhabitants	25.6	29.9
2,000–4,999 inhabitants	6.9	7.9
5,000–9999 habitants	5.6	6.6
10,000–19,999 habitants	5.3	5.8
20,000–49,999 habitants	6.5	6.7
50,000–99,999 habitants	7.1	7.3
100,000–199,999 habitants	6.0	4.7
200,000–1,999,999 habitants	21.4	21
City of Paris	15.4	10.0
*Region*		
Rhône Alpes Auvergne (South-East)	11.7	12.8
Bretagne, Loire et Centre (North-West)	13.2	16.9
Bourgogne-Franche Comté (North-East)	3.7	4.6
Grand Est (North-East)	9.1	9.6
Hauts de France (North)	10.6	9.3
Ile-de-France (great Paris)	17.8	14.6
Nouvelle Aquitaine (South-West)	9.1	10.3
Normandie (North-West)	4.6	5.6
Occitanie (South-West)	9.1	9.0
Provence-Alpes-Côte d’Azur, Corse (South-East)	8.4	7.1
Outre mer (Overseas territories)	2.5	-
*Global Activity Limitation Indicator*		
Not limited	71.7	73.3
Limited, not severely	17.4	18.8
Limited, severely	10.9	7.9
*Self perceived Health*		
Very good—Good	66.8	65.2
Fair	22.6	26.5
Bad—Very bad	10.6	8.3
*ADL/IADL*		
No limitation	86.9	–
Limitation in < 3 ADLs and < 2 IADLs	7.1	–
Limitation in ≥ 3 ADLs or ≥ 2 IADLs	6.0	–
Mean SF-12 total score (SEM)*	71.5 (0.2)	–
Followed up**	–	96.9
Deceased 2010–2014		2.1
New limitation at 4 years***		16.6
New health deterioration at 4 years ****		16.9

* Missing in 9,808 subjects

† Household incomes categorized according to tax bracket, split into three approximately equal parts (tertiles) in each survey. Subjects could refuse to provide their tax bracket

** Follow-up was scheduled in 7,727 subjects

*** Limitation in 2014 in those not limited in 2010 (N = 2,101)

**** Health graded less than good in 2014 in those having good/very good health in 2010 (N = 1,886)

*Abbreviations*. ADL: activities of daily living; IADL: instrumental activities of daily living; SF-12: Medical Outcomes Study Short-Form 12-Item Health Survey; SEM: standard error of the mean.

### Prevalence of multimorbidity across gender and age groups and associations with health status indicators

Among women 1-year multimorbidity prevalence was 34.4% (95% confidence interval [CI]: 33.2–35.6%) and lifetime multimorbidity prevalence was 42.5% (95% CI: 41.2–43.8%), whereas the corresponding figures for men were 26.0% (95% CI: 24.9–27.1%) and 35.1% (95% CI: 33.7–36.5%), respectively.

**Fig 1:**
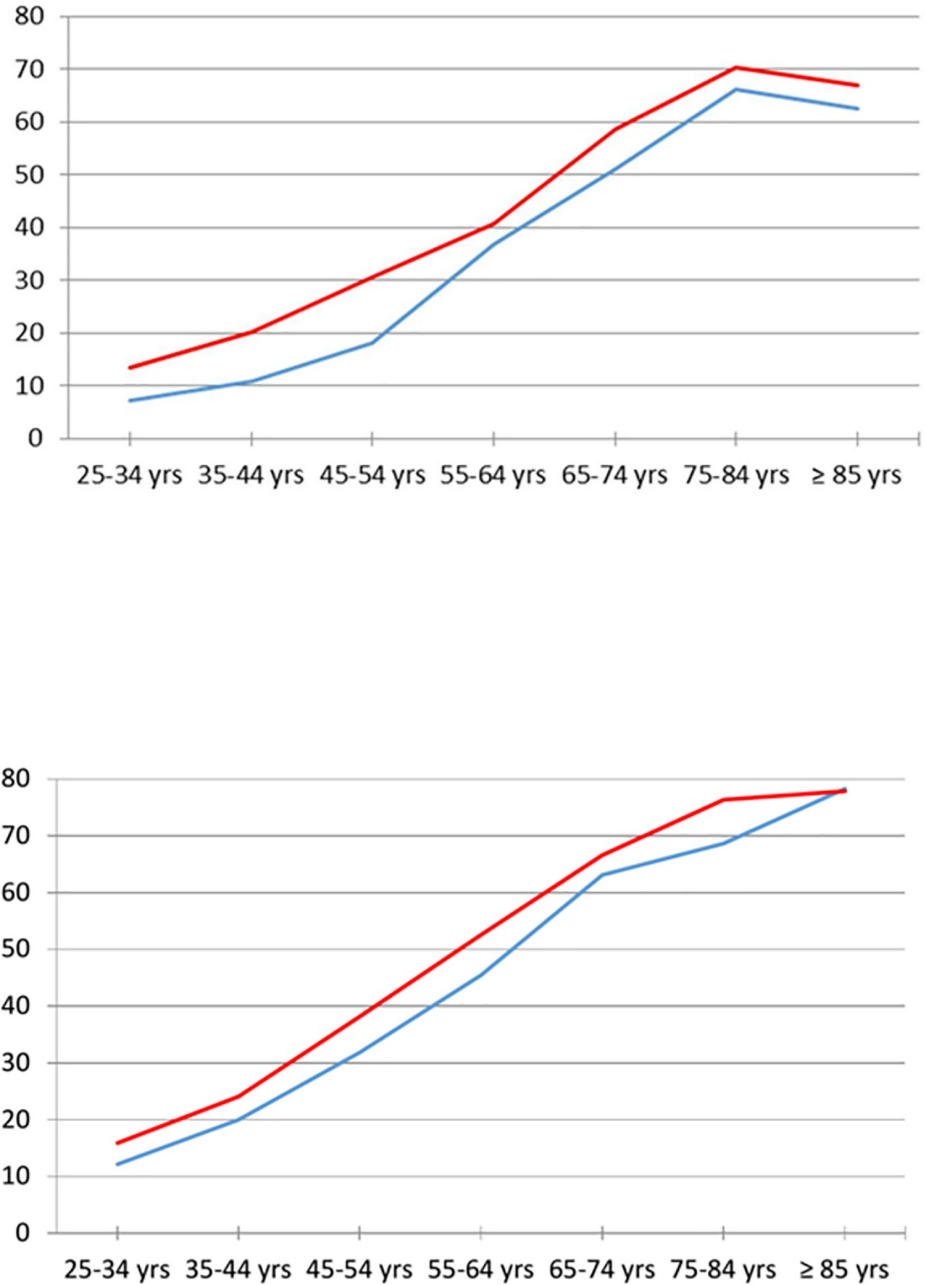
Age- and gender-specific prevalence of multimorbidity. Upper panel: 1 yr prevalence, Lower panel: lifetime prevalence. Ordinates represent frequencies in percentages. Red and blue lines indicate women (red) and men (blue).

For both 1-year and lifetime frames, the burden of chronic conditions and multimorbidity increased steadily with age and stabilized after 75 yrs (Table 2). The greatest increases in the 1-year multimorbidity prevalence were observed at 55–64 years in men, and 65–74 years in women. The prevalence of multimorbidity in young women was almost twice that of young men, but differences across gender were smaller for older categories. The difference between men and women in the prevalence of lifetime multimorbidity was smaller and the trends more linear. Overall women experienced multimorbidity 23–31% more frequently and 5–15 yrs earlier than men (Fig 1).

**Table 2:** Weighted frequencies of subjects presenting with ≥ 1, ≥ 2, ≥ 3 and ≥ 4 conditions in ESPS and HSM surveys. All estimates are weighted to represent French population estimates.

		1-year timeframe (ESPS Survey)							Lifetime timeframe (HSM Survey)						
		25–34 yrs	35–44 yrs	45–54 yrs	55–64 yrs	65–74 yrs	75–84 yrs	≥ 85 yrs	25–34 yrs	35–44 yrs	45–54 yrs	55–64 yrs	65–74 yrs	75–84 yrs	≥ 85 yrs
Men	≥1 condition	22.8	30.0	36.7	53.9	64.0	76.6	75.1	38.6	52.0	61.4	75.3	86.0	88.4	91.8
** **	≥2 conditions	7.1	10.9	18.2	36.7	51.0	66.2	62.4	12.2	20.0	31.9	45.5	63.1	68.7	78.3
	≥3 conditions	2.5	4.4	9.3	22.3	35.2	52.4	47.4	5.3	9.1	14.6	23.9	42.1	50.5	59.0
	≥4 conditions	1.1	1.9	4.2	13.2	21.9	38.7	38.2	1.8	3.6	8.3	12.8	25.6	32.5	36.1
Women	≥1 condition	33.2	38.7	48.0	58.2	71.2	78.9	78.9	42.5	52.8	65.4	79.8	86.6	93.1	91.6
** **	≥2 conditions	13.3	20.2	30.7	40.6	58.7	70.3	67.0	15.9	24.1	38.2	52.6	66.6	76.4	77.9
** **	≥3 conditions	5.9	8.4	19.3	27.3	43.1	55.6	57.8	6.5	10.3	20.8	34.5	44.0	56.9	58.1
** **	≥4 conditions	2.6	2.9	10.8	18.1	31.9	40.4	47.8	2.2	5.1	11.0	21.8	28.3	41.3	44.2

For both genders and most health indicators, the associations with age were increasingly weaker across (increasing) age categories, except for the health indicators “new limitation” and “new health deterioration” (Table 3).

**Table 3:** Associations of multimorbidity with health outcomes according to age and gender as estimated in multiple logistic regression models.

	1-year multimorbidity (ESPS Survey)						Lifetime multimorbidity (HSM Survey)					
	*Men*			*Women*			*Men*			*Women*		
	25–54 yrs	55–74 yrs	≥ 75 yrs	25–54 yrs	55–74 yrs	≥ 75 yrs	25–54 yrs	55–74 yrs	≥ 75 yrs	25–54 yrs	55–74 yrs	≥ 75 yrs
GALI limited, severely*	8.56 (5.51–13.31)	9.16 (5.22–16.07)	12.37 (4.69–32.61)	8.43 (5.28–13.46)	9.95 (5.39–18.38)	9.57 (3.88–23.58)	8.40 (6.31–11.17)	6.90 (5.11–9.30)	5.95 (3.89–9.10)	12.71 (9.59–16.85)	8.49 (6.31–11.44)	6.56 (4.61–9.32)
GALI limited, not severely*	5.14 (3.85–6.88)	4.64 (3.39–6.36)	2.66 (1.46–4.85)	5.87 (4.57–7.54)	6.30 (4.36–9.10)	2.62 (1.37–5.02)	5.62 (4.35–7.27)	4.65 (3.57–6.05)	4.27 (2.68–6.82)	6.49 (5.21–8.08)	5.26 (4.06–6.81)	3.97 (2.76–5.71)
Bad or very bad health*	12.70 (8.06–20.00)	11.49 (6.25–21.10)	8.57 (3.50–20.98)	23.90 (14.77–38.67)	16.15 (8.07–32.32)	11.06 (4.05–30.17)	10.83 (7.72–15.21)	9.86 (7.20–13.50)	6.41 (3.94–10.42)	14.73 (10.94–19.84)	12.14 (9.09–16.22)	8.20 (5.69–11.83)
Fair health*	4.57 (3.54–5.90)	4.60 (3.51–6.02)	2.89 (1.66–5.03)	5.71 (4.63–7.03)	4.46 (3.33–5.98)	2.71 (1.53–4.79)	5.42 (4.27–6.89)	4.08 (3.19–5.23)	2.97 (1.91–4.61)	5.66 (4.57–7.01)	4.72 (3.72–5.98)	3.42 (2.39–4.89)
Limitation in ≥ 3 ADLs or ≥ 2 IADLs*							8.96 (6.22–12.90)	4.04 (2.72–6.01)	3.29 (2.10–5.17)	10.99 (8.15–14.80)	6.27 (4.30–9.13)	3.90 (2.82–5.40
Limitation in < 3 ADLs and < 2 IADLs*							4.56 (3.16–6.58)	2.80 (1.84–4.25)	2.69 (1.61–4.49)	7.28 (5.17–10.24)	3.44 (2.44–4.85)	2.66 (1.76–4.02
Death between 2010 and 2014	5.89 (1.61–21.54)	1.01 (0.48–2.12)	0.79 (0.42–1.50)	2.26 (0.53–9.64)	1.06 (0.35–3.21)	3.79 (1.28–11.20)						
New limitation**	2.08 (1.09–3.97)	2.37 (1.25–4.50)	2.10 (0.49–9.01)	4.49 (2.61–7.70)	4.30 (2.20–8.40)	14.18 (1.66–121.09)						
New health deterioration***	2.34 (1.20–4.58)	3.31 (1.63–6.73)	3.34 (0.87–12.86)	2.39 (1.38–4.13)	4.25 (2.15–8.43)	5.04 (0.92–27.54)						

Multimorbidity defined by ≥ 2 conditions present during the previous 12 months (ESPS Survey) or across lifetime (HSM Survey). Odds ratios and 95% confidence intervals.

* Polytomous logistic regression using “no limitation” or “good or very good health” or “no limitation in ADL/IADL” as the reference category. Odds ratios are unadjusted.

** Limitation, severe or not in 2014 in subjects who were not limited in 2010

*** Health graded less than good in 2014 in subjects with good/very good health in 2010.

Stronger associations with perceived health and new limitations were observed in women and with mortality in men.

### Conditions involved in multimorbid associations across age and gender groups

**Fig 2:**
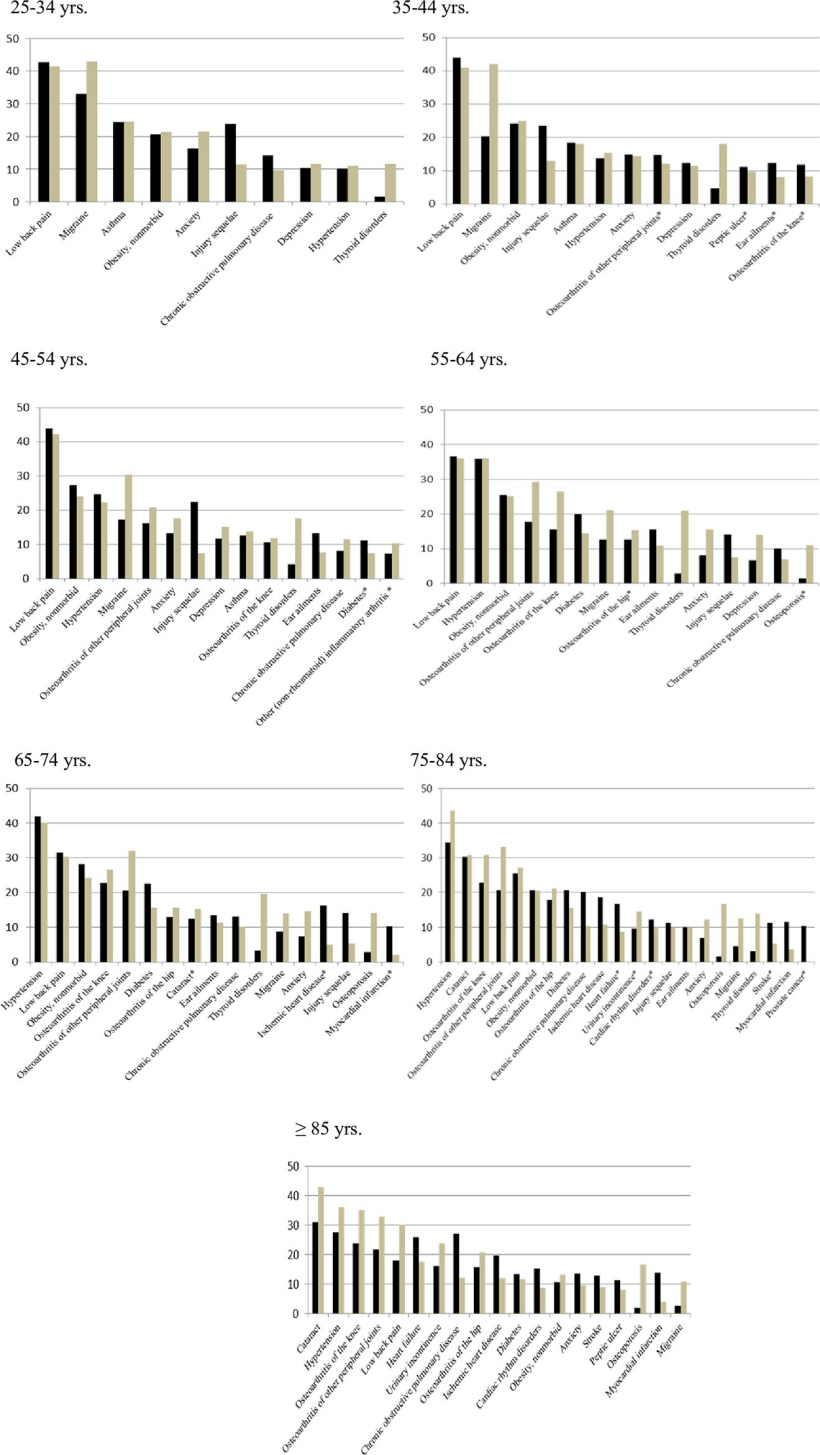
Top most frequent (> 10%) conditions in the 14 gender and age groups of multimorbid subjects in the HSM survey. Ordinates represent frequencies in percentages. Conditions are ranked according to the median percentage of frequencies for both genders. Asterisk indicates a condition making its first entry in the top frequent ones at that age category.

Fig 2 presents the most frequent conditions involved in lifetime multimorbidity associations across gender and age (frequencies for all conditions involved are reported in S1 Table). The presence of these conditions was consistent with their own usual patterns of occurrence across age and gender. However, the differences in rankings observed across gender and age groups were large and only a few conditions (low back pain, hypertension, obesity and anxiety) remained in the top rankings for all gender and age categories.

The most frequent dyads and triads of chronic conditions along with their distribution across gender and age categories in HSM survey are presented in S2 Table. These combinations included most frequently hypertension, low back pain, obesity, osteoarthritis (knee, hip, or other peripheral joints), migraine, diabetes, anxiety, depression and ear ailments, and their frequencies increased with age with the exception of those involving migraine, thyroid disorders, anxiety and depression which predominantly affected young and middle-aged subjects, notably women. Differences in terms of multimorbid components also concerned older women, who were more effected by osteoporosis and osteoarthritis (especially other than hip or knee) than men. In the latter, while injury sequelae were more markedly prevalent in young individuals, they were still present in older persons. Furthermore, COPD and ischemic cardiovascular disease tended to be more prevalent at older ages (≥ 65 yrs) in men than in women.

### Multimorbidity burden across educational, socioeconomic and geographic groups

Table 4 presents the risk of having more than 1, 2, 3 and 4 chronic conditions associated with education level, occupation and household income. The inverse dose-response relationship of accumulated conditions with education level was especially strong and consistent: the odds-ratios reached or even exceeded 2 (for 4 conditions) for lower and middle education levels in both surveys. The associations of accumulated conditions with manual worker occupation, and to a lesser extent with intermediate professionals (e.g., teachers, middle managers), and lower thirds of household income, are also consistent across surveys but dose-response relationships were less marked with these indicators once educational level had been adjusted for.

**Table 4:** Age and gender adjusted risk of having more than 1, 2, 3 and 4 conditions, associated with each separate (upper panel) or with the three socioeconomic status indicators (education level, occupation, and household income) (lower panel) as estimated in multiple binary logistic regression (full model).

		**1-year time frame (ESPS Survey)**							
		Education (Ref: Tertiary)		Occupation (Ref: Manager, professional)			Household incomes (Ref: Not provided*)		
		Secondary	Less than secondary	Middle manager, teacher	No occupation or studying	Manual worker	Upper third	Middle third	Lower third
A. Separate models for each indicator adjusted for age and gender	≥1 condition	1.08 (0.98–1.19)	1.02 (0.92–1.14)	**1.19 (1.06–1.33)**	0.91 (0.73–1.14)	1.10 (0.98–1.23)	**4.86 (4.34–5.44)**	**5.19 (4.62–5.83)**	**6.32 (5.57–7.16)**
	≥2 conditions	**1.25 (1.11–1.41)**	**1.24 (1.10–1.41)**	**1.31 (1.15–1.49)**	1.14 (0.88–1.47)	**1.23 (1.08–1.39)**	**3.10 (2.72–3.52)**	**3.98 (3.49–4.53)**	**4.67 (4.09–5.34)**
	≥3 conditions	**1.40 (1.21–1.63)**	**1.44 (1.23–1.68)**	**1.37 (1.17–1.60)**	**1.38 (1.04–1.83)**	**1.38 (1.19–1.60)**	**2.13 (1.83–2.47)**	**3.01 (2.60–3.49)**	**3.52 (3.04–4.08)**
	≥4 conditions	**1.82 (1.48–2.23)**	**1.98 (1.60–2.43)**	**1.64 (1.34–1.99)**	**1.82 (1.30–2.56)**	**1.83 (1.52–2.19)**	**1.71 (1.42–2.07)**	**2.69 (2.26–3.20)**	**3.61 (3.05–4.28)**
B. Full combined model adjusted for age, gender and including all three indicators	≥1 condition	1.04 (0.93–1.17)	**1.01 (0.88–1.16)**	1.09 (0.96–1.24)	0.92 (0.73–1.17)	0.97 (0.85–1.12)	**4.81 (4.29–5.40)**	**5.16 (4.59–5.80)**	**6.36 (5.60–7.23)**
	≥2 conditions	**1.17 (1.02–1.34)**	**1.17 (1.01–1.36)**	1.13 (0.98–1.30)	1.03 (0.80–1.33)	0.97 (0.84–1.13)	**3.14 (2.75–3.59)**	**3.93 (3.45–4.48)**	**4.64 (4.05–5.32)**
	≥3 conditions	**1.24 (1.05–1.47)**	**1.25 (1.04–1.50)**	1.16 (0.98–1.37)	1.21 (0.91–1.61)	1.06 (0.89–1.26)	**2.22 (1.90–2.59)**	**2.97 (2.57–3.45)**	**3.45 (2.97–4.00)**
	≥4 conditions	**1.46 (1.17–1.83)**	**1.50 (1.18–1.90)**	**1.31 (1.06–1.62)**	**1.49 (1.05–2.10)**	**1.28 (1.03–1.58)**	**1.89 (1.56–2.30)**	**2.65 (2.22–3.16)**	**3.43 (2.88–4.07)**
		**Lifetime frame (HSM Survey)**							
		Education (Ref: Tertiary)		Occupation (Ref: Manager, professional)			Household incomes (Ref: Not provided*)		
		Secondary	Less than secondary	Middle manager, teacher	No occupation or studying	Manual worker	Upper third	Middle third	Lower third
A. Separate models for each indicator adjusted for age and gender	≥1 condition	**1.33 (1.17–1.50)**	**1.76 (1.52–2.03)**	**1.27 (1.09–1.47)**	1.12 (0.71–1.78)	**1.55 (1.33–1.79)**	**1.22 (0.99–1.49)**	**1.23 (1.00–1.51)**	**1.61 (1.31–1.98)**
	≥2 conditions	**1.43 (1.27–1.62)**	**2.04 (1.78–2.33)**	**1.55 (1.34–1.79)**	1.39 (0.87–2.24)	**1.96 (1.71–2.26)**	**1.18 (0.98–1.42)**	**1.37 (1.14–1.64)**	**1.84 (1.54–2.20)**
	≥3 conditions	**1.39 (1.20–1.61)**	**2.06 (1.77–2.39)**	**1.48 (1.26–1.75)**	1.69 (0.94–3.03)	**2.02 (1.72–2.37)**	0.99 (0.82–1.21)	**1.20 (1.00–1.45)**	**1.72 (1.43–2.06)**
	≥4 conditions	**1.45 (1.22–1.72)**	**2.18 (1.83–2.60)**	**1.50 (1.25–1.81)**	**2.15 (1.10–4.19)**	**2.14 (1.78–2.57)**	0.81 (0.65–1.01)	**1.27 (1.03–1.56)**	**1.63 (1.34–1.98)**
B. Full combined model adjusted for age, gender and including all three indicators	≥1 condition	**1.24 (1.08–1.42)**	**1.56 (1.31–1.85)**	1.14 (0.97–1.34)	0.95 (0.60–1.51)	**1.25 (1.05–1.49)**	**1.38 (1.12–1.70)**	1.22 (0.99–1.50)	**1.48 (1.20–1.83)**
	≥2 conditions	**1.23 (1.08–1.42)**	**1.60 (1.36–1.88)**	**1.32(1.13–1.55)**	1.10 (0.69–1.76)	**1.48 (1.25–1.74)**	0.72 (0.59–0.87)	0.97 (0.86–1.10)	**1.18 (1.04–1.35)**
	≥3 conditions	**1.17 (1.00–1.38)**	**1.52 (1.27–1.81)**	**1.25 (1.04–1.49)**	1.28 (0.72–2.29)	**1.47 (1.22–1.78)**	1.17 (0.96–1.44)	**1.21 (1.00–1.46)**	**1.55 (1.28–1.86)**
	≥4 conditions	**1.16 (0.96–1.40)**	**1.49 (1.21–1.83)**	1.19 (0.97–1.46)	1.54 (0.79–2.98)	**1.46 (1.18–1.80)**	0.96 (0.76–1.20)	**1.29 (1.05–1.58)**	**1.47 (1.21–1.80)**

Odds ratios and 95% confidence intervals. One-year timeframe and life timeframe. For the three indicators, the category with the lowest risk was selected as the reference group. Since reference categories correspond to the highest status (except for household income*), comparing the ordered categories of indicators to the reference categories allows for the direct assessment of the dose-effect relationship.

* No provided household income was generally associated with the lowest risk of chronic condition (whatever the threshold) and therefore taken as the reference category.

Table 5 presents the results of the analysis of socioeconomic differences conducted at the level of multimorbid disease combinations. The lowest levels of education and income were independently associated with many dyads and triads, notably those including obesity, diabetes, low back pain and migraine. Similarly manual labour, and to a lesser extent intermediate profession employment categories appeared to be associated with various dyads and triads including low back pain, injury sequelae, COPD, anxiety and osteoarthritis.

**Table 5:** Risk of a given dyad or triad associated with the three socioeconomic status indicators (education level, occupation, and household income) as estimated in multiple binary logistic regression models including age, gender, and all three sociodemographic variables.

	Education (Ref: Tertiary)		Occupation (Ref: Manager, professional)			Household incomes (Ref: Not provided*)		
	Less than secondary	Secondary	Manual worker	Middle manager, teacher	No occupation or studying	Lower third	Middle third	Upper third
***I*. *Dyads***								
***Any dyad***	**1.60**	**1.23**	**1.48**	**1.32**	1.10	**1.65**	**1.35**	**1.39**
Hypertension-Low back pain	**1.61**	1.40	1.10	1.02	0.65	1.38	1.39	1.17
Obesity, nonmorbid-Hypertension	**1.88**	1.33	1.14	1.07	1.12	**1.50**	**1.59**	1.16
Migraine-Low back pain	1.15	0.99	**2.01**	**1.84**	2.04	**1.55**	1.27	1.46
Osteoarthritis of other peripheral joints-Low back pain	1.41	1.33	**1.57**	1.46	1.31	1.22	1.19	1.05
Osteoarthritis of the knee-Low back pain	**7.22**	2.56	0.80	0.70	0.26	0.77	0.79	0.51
Hypertension-Osteoarthritis of other peripheral joints	1.44	1.09	1.13	0.99	1.57	1.34	1.19	0.82
Osteoarthritis of the knee-Osteoarthritis of other peripheral joints	1.27	0.95	**2.12**	**2.02**	1.27	1.25	1.08	0.89
Diabetes-Hypertension	**1.95**	1.30	1.15	0.98	1.07	1.00	1.01	0.83
Hypertension-Osteoarthritis of the knee	**1.67**	1.29	1.18	1.00	0.91	1.06	1.01	0.89
Obesity, nonmorbid-Low back pain	**2.15**	1.67	1.47	1.32	0.50	1.43	**1.48**	1.00
Anxiety-Low back pain	0.92	0.78	**2.00**	1.48	2.86	**2.27**	**1.73**	1.02
Osteoarthritis of the hip-Osteoarthritis of the knee	1.62	1.18	1.34	1.19	0.36	1.25	1.21	0.84
Obesity, nonmorbid-Osteoarthritis of the knee	**2.02**	1.43	1.83	1.45	2.29	0.99	0.92	0.70
Osteoarthritis of the hip-Low back pain	1.52	1.22	1.27	1.16	0.69	1.19	1.35	0.87
COPD-Osteoarthritis of other peripheral joints	1.09	0.70	**4.17**	**4.05**	2.23	1.70	1.23	0.77
Low back pain-Injury sequelae	0.73	0.97	**2.61**	**2.16**	2.53	**3.41**	**2.70**	**1.95**
Diabetes-Obesity, nonmorbid	**2.08**	1.14	1.45	1.15	0.37	0.99	0.99	0.73
Osteoarthritis of the hip-Osteoarthritis of other peripheral joints	1.10	0.81	1.27	1.15	0.46	1.56	1.68	0.84
Obesity, nonmorbid-Osteoarthritis of other peripheral joints	**2.48**	1.63	**2.13**	1.69	1.83	1.34	1.01	0.88
Cataract-Hypertension	1.31	1.12	0.80	0.81	1.11	1.19	1.10	1.09
Migraine-Anxiety	**2.09**	0.99	**2.77**	**2.35**	1.60	1.47	0.91	0.92
Thyroid disorders-Low back pain	**1.73**	1.22	1.13	1.58	0.33	1.49	**1.79**	**1.85**
Migraine-Osteoarthritis of other peripheral joints	**2.52**	1.64	1.08	0.85	1.22	1.29	1.08	1.05
Migraine-Hypertension	1.46	1.14	1.31	1.11	1.41	**1.70**	1.02	0.64
Hypertension-Osteoarthritis of the hip	1.65	1.03	1.53	1.76	0.57	1.38	1.09	0.84
Depression-Low back pain	1.23	1.17	1.63	1.42	1.33	**2.34**	1.47	0.88
Ear ailments-Low back pain	1.60	1.54	0.69	0.81	2.37	1.29	1.27	1.04
Asthma-Low back pain	1.05	0.97	1.84	1.49	1.07	1.24	0.91	1.32
Depression-Anxiety	**1.68**	1.44	**2.18**	1.54	2.14	1.14	0.62	**0.38**
Other inflammatory arthritis-Low back pain	1.29	1.11	**2.40**	1.86	1.75	**2.81**	**2.95**	1.95
Cataract-Low back pain	1.19	1.04	1.50	1.23	0.06	1.48	1.30	1.38
COPD-Low back pain	1.11	0.93	**2.69**	**2.54**	0.75	1.64	1.25	0.90
Thyroid disorders-Hypertension	1.26	1.16	1.62	**1.90**	4.98	1.41	1.54	1.22
Diabetes-Low back pain	**3.52**	1.69	0.92	0.85	0.89	1.05	0.90	0.83
Anxiety-Hypertension	1.52	1.28	1.69	1.20	1.72	**2.18**	1.47	1.02
Anxiety-Osteoarthritis of other peripheral joints	1.07	0.70	**3.56**	**2.53**	**5.01**	**1.64**	1.17	1.25
Cataract-Osteoarthritis of other peripheral joints	1.07	0.73	0.80	0.72	1.19	1.30	1.08	0.75
Migraine-Osteoarthritis of the knee	1.70	1.15	**2.75**	**2.22**	0.77	1.33	0.99	0.80
Peptic ulcer-Low back pain	**2.25**	1.16	1.83	**2.05**	1.56	**2.16**	1.66	1.52
Migraine-Injury sequelae	1.39	1.26	1.85	1.33	1.95	**1.69**	1.35	1.34
Depression-Migraine	**1.88**	1.23	**2.54**	1.90	2.28	**2.16**	1.32	1.08
Ear ailments-Hypertension	1.46	1.42	1.73	1.36	0.49	**2.19**	**2.02**	1.47
Cataract-Osteoarthritis of the knee	0.91	0.79	0.85	0.81	1.46	1.22	0.96	0.92
Hypertension-COPD	1.35	1.04	1.27	0.97	0.39	**1.72**	1.28	0.73
Hypertension-Injury sequelae	1.42	1.10	1.41	1.12	0.82	1.26	1.07	0.93
Diabetes-Osteoarthritis of the knee	**2.86**	1.79	0.85	0.65	**0.10**	0.99	1.01	**0.43**
Hypertension-Ischemic heart disease	0.86	0.90	1.64	1.05	1.54	1.14	0.83	0.79
Anxiety-Osteoarthritis of the knee	1.18	0.64	**4.25**	**3.40**	**6.75**	**1.56**	1.21	0.71
Thyroid disorders-Osteoarthritis of other peripheral joints	1.59	0.94	1.09	1.03	0.23	1.14	1.53	1.18
Osteoarthritis of other peripheral joints-Injury sequelae	1.31	0.88	1.78	1.48	**<0.001**	1.06	1.01	1.01
Low back pain-Urinary incontinence	0.89	1.07	1.82	1.83	3.84	**2.12**	**1.85**	1.68
Hypertension-Cardiac rhythm disorders	1.40	1.32	0.88	0.76	0.67	1.32	1.09	0.66
Cardiac rhythm disorders-Low back pain	0.50	0.56	**3.79**	**3.23**	**6.56**	**1.99**	**1.92**	**2.04**
Obesity, nonmorbid-Migraine	**5.17**	**3.08**	0.58	0.62	1.00	**1.88**	1.11	0.83
COPD-Asthma	1.64	1.05	1.86	1.19	**0.10**	1.24	0.75	1.54
Migraine-COPD	**2.53**	1.82	2.03	1.72	**0.09**	1.57	0.98	1.10
Osteoarthritis of the knee-Injury sequelae	1.05	1.23	2.02	**1.90**	1.28	**2.29**	1.90	1.11
Low back pain-Osteoporosis	0.88	0.98	1.28	1.51	0.63	1.49	1.00	1.18
Diabetes-Osteoarthritis of other peripheral joints	**2.28**	1.19	1.81	1.62	0.98	1.14	1.07	0.67
Obesity, nonmorbid-Asthma	1.39	0.64	1.90	2.03	**0.07**	1.70	0.89	1.08
Migraine-Ear ailments	1.64	1.11	1.54	1.49	0.71	1.21	0.96	0.82
Hypertension -Asthma	1.27	0.98	1.21	1.05	1.24	**1.95**	1.28	0.87
Hypertension-Heart failure	1.36	1.04	1.19	0.78	0.51	**2.63**	**2.04**	1.60
Thyroid disorders-Obesity, nonmorbid	**2.38**	**1.96**	1.01	1.12	0.16	1.54	1.45	1.17
Depression-Osteoarthritis of other peripheral joints	1.39	1.07	1.23	1.04	2.63	**1.87**	1.04	0.79
Hypertension-Urinary incontinence	1.58	1.68	1.18	0.85	0.75	1.40	1.44	1.26
Obesity, nonmorbid-Osteoarthritis of the hip	**2.35**	1.48	1.66	1.49	0.54	1.55	1.32	1.16
Migraine-Osteoarthritis of the hip	**8.85**	**5.77**	1.18	0.73	**0.13**	0.73	0.57	**0.30**
Other inflammatory arthritis-Osteoarthritis of the knee	2.08	1.75	0.96	1.04	0.03	1.66	1.23	0.88
Depression-Hypertension	1.20	0.99	1.28	1.37	2.24	**1.85**	1.00	1.03
Obesity, non morbid-Anxiety	1.82	1.12	**3.02**	2.44	0.35	**2.01**	**2.03**	0.77
Other inflammatory arthritis-Hypertension	1.06	1.06	1.30	1.10	**0.11**	1.71	1.46	1.43
Ear ailments-Osteoarthritis of other peripheral joints	**1.55**	2.09	2.05	1.35	2.00	1.61	1.58	1.49
Migraine-Asthma	**2.56**	**2.20**	1.14	0.93	0.79	1.91	1.11	1.38
Obesity, nonmorbid-Injury sequelae	1.16	1.38	1.69	1.31	**<0.001**	1.20	0.65	0.65
COPD-Osteoarthritis of the knee	1.70	1.12	**3.49**	**2.29**	8.02	1.48	1.45	1.20
Migraine-Peptic ulcer	**5.08**	**2.60**	1.13	1.44	1.59	1.09	0.87	1.07
Thyroid disorders-Migraine	**3.01**	1.24	1.35	1.38	**0.08**	1.51	1.33	1.36
Ischemic heart disease-Low back pain	0.95	0.81	**2.52**	2.06	1.34	1.50	1.08	1.53
Hypertension-Peptic ulcer	1.08	0.76	1.96	1.88	1.98	**1.97**	0.96	0.87
Obesity, non morbid-Ear ailments	2.57	1.11	2.01	2.04	0.14	1.00	1.26	0.70
Ear ailments-Osteoarthritis of the knee	1.19	0.91	2.13	**2.21**	**<0.001**	1.29	1.16	1.35
Osteoarthritis of other peripheral joints-Osteoporosis	0.89	0.71	1.40	1.86	1.71	1.66	1.37	1.68
Hypertension-Osteoporosis	1.55	1.81	**3.06**	**3.81**	**7.61**	1.86	1.14	1.95
Anxiety-Osteoarthritis of the hip	0.98	0.49	2.29	1.91	**0.10**	0.94	1.09	0.42
Depression-Osteoarthritis of the knee	1.44	1.11	1.90	1.31	2.91	**2.32**	1.36	1.11
Osteoarthritis of other peripheral joints-Urinary incontinence	0.87	0.70	1.90	1.13	**0.22**	1.43	1.56	1.33
Obesity, nonmorbid-COPD	1.61	0.83	1.51	1.22	4.17	1.66	1.38	1.02
***II*. *Triads***								
***Any triad***	***1*.*52***	*1*.*17*	***1*.*47***	***1*.*25***	*1*.*28*	***1*.*55***	***1*.*21***	*1*.*17*
Osteoarthritis of the hip-Osteoarthritis of the knee-Osteoarthritis of other peripheral joints	1.44	1.14	1.84	1.68	**<0.001**	1.61	1.43	1.06
Osteoarthritis of the hip-Osteoarthritis of the knee-Low back pain	1.26	1.01	1.40	1.10	0.62	1.18	1.27	0.57
Osteoarthritis of the knee-Osteoarthritis of other peripheral joints-Low back pain	1.33	1.07	**3.83**	**3.42**	0.25	0.98	0.87	0.90
Hypertension-Osteoarthritis of the knee-Low back pain	**2.72**	2.01	1.40	1.17	**<0.001**	1.09	1.25	0.69
Migraine-Osteoarthritis of the knee-Low back pain	**9.18**	**7.59**	3.40	1.93	**0.18**	1.09	0.75	0.46
Obesity, nonmorbid-Hypertension-Low back pain	1.14	0.99	1.50	1.38	0.14	1.65	1.63	0.87
Diabetes-Obesity, nonmorbid-Hypertension	**2.52**	1.67	1.82	1.56	1.01	1.38	1.62	1.04
Osteoarthritis of the hip-Osteoarthritis of other peripheral joints-Low back pain	1.65	1.38	1.62	1.32	0.53	1.22	1.27	0.72
Obesity, nonmorbid-Hypertension-Osteoarthritis of the knee	1.41	1.07	1.93	1.53	**<0.001**	0.95	0.92	0.59
Anxiety-Osteoarthritis of the knee-Low back pain	1.72	0.96	**9.20**	**6.45**	0.87	1.42	1.08	0.69
Migraine-Anxiety-Low back pain	1.53	0.84	2.59	1.71	0.35	1.51	1.08	0.78
Hypertension-Osteoarthritis of other peripheral joints-Low back pain	1.24	0.95	1.24	0.99	0.80	1.59	1.47	0.77

Analyses were conducted within the lifetime timeframe (HSM Survey). Odds ratios are presented. The figures in bold indicate statistical significance.

* see Table 4.

On the contrary, there were no relationship between multimorbidity and the size of the urban unit and only a weak and inconsistent relationship between multimorbidity and geographic area (region) in both surveys (except for Haut de France region), once the effects of the three socioeconomic indicators had been adjusted for (S3 Table). Geographical and territorial differences are explained in large part by educational and socioeconomic factors.

## Discussion

The analysis of two large representative general population samples with cross-sectional and longitudinal data on numerous chronic conditions and a broad range of health indicators allowed us to characterize as finely as possible the burden of multimorbidity in France. These results especially highlight the diverse and changeable components and impacts of multimorbidity across gender and age, and evidence strong associations with socioeconomic factors, notably educational level, for which causality appears likely. These results, together with those obtained regarding impacts, interaction and etiological patterns of combinations in the same population [10], shed new light on multimorbidity issues and reveal their full implications in terms of surveillance and prevention.

### Gender differences

Our results showing that women experienced more (23–31%)–but slightly different–chronic conditions than men, and that they experienced them earlier (5–15 yrs), add to previous knowledge which mainly concerned quantitative counts [1, 2]. These results are consistent with the repeated observation of the higher prevalence of chronic conditions among women, especially young and middle-aged women in all regions of the world [13]. As to the widely believed assumption that women more readily report illness, evidence is contradictory and unsupportive [14, 15]. On the contrary, the evidence is stronger for women seeking more help for healthcare issues than men [16, 17].

Differences in terms of multimorbid components concerned both young women–where migraine, anxiety and thyroid disorders are more prevalent–and older women, who are more effected by osteoporosis and osteoarthritis than men. On the contrary, older men are more affected by COPD and ischemic cardiovascular disease than women. The different nature of the constituent diseases across genders explains that multimorbidity is more strongly associated with mortality for men, especially at younger age, than for women, who had stronger associations with functional limitation and poorer reported health status.

Altogether, these differences underline the need for separate assessment of multimorbidity according to gender. This is in line with recommendations regarding other health indicators such as mortality and life expectancy [18] or patient reported outcomes [19] and quality of life [20].

### Age differences

This study found firm evidence for the increases in multimorbidity with age from early midlife onwards: 35–44 yrs in women and 55–64 yrs in men [2]. These increases appeared more linear with lifetime multimorbidity than with 1-yr multimorbidity, but in all cases the prevalence stabilized after 75 yrs. This study also showed that most common associations of morbid conditions varied with aging: while the prevalence of most chronic conditions increased with age (with different kinetics), it decreased for others, such as migraine or asthma). In addition, the association of multimorbidity with most health status indicators weakened (in terms of odds ratios) with increasing age; this result is novel and merits further investigation. Contrary to the risk ratios, odds ratios are not bound by mathematically defined ceilings, and the increasing basic levels of risks of activity limitation or poorer perceived health with age cannot explain why these associations weaken with age [21, 22]. Several factors may be highlighted instead: survival and successful aging could be associated with greater resilience to chronic disease, and older persons would be less impacted than younger individuals (older individuals living in nursing homes, probably the most severely impacted by multimorbidity, were not included in this study). Other health-related concepts such as disability and frailty [23, 24] may also become more important over time than morbidity *per se*. Whatever the case, multimorbidity is definitely less of a problem in very old persons than their middle-aged counterparts in terms of the impact on health outcomes.

### Socioeconomic and territorial inequalities

Our finding that multimorbidity is strongly and independently associated with three socioeconomic indicators (education, occupation and income) but less consistently with geographic factors (size of the urban unit and region) adds to disparate and scattered knowledge from ecological studies and studies mostly measuring socioeconomic status at a collective level, using the surrogate variable “deprived areas”, which mixes social and territorial dimensions [25–32]. The socioeconomic indicator with the strongest inverse dose-response relationship with the number of chronic diseases and many dyads and triads (especially those including obesity, diabetes and low back pain) was educational level. This result is consistent with the systematic review of Pathirana and Jackson [9] and the results of several studies conducted in Europe during the last decade [33–38]. Insofar as chronology is unambiguous (education usually precedes chronic condition occurrence), there is thus consistent evidence in favor of a causal relationship between education and multimorbidity. Such a causal relationship has increasingly been advocated between education and health in general [39–41]. Occupation and income have also been strongly associated with multimorbidity and various dyads and triads, but the related dose-response relationships were often less marked as mediated effects may probably be more complex [38, 42, 43]. As regards geographical and territorial inequalities, only the ‘Haut de France’ region was consistently associated with a higher risk of multimorbidity in our study. This deindustrialized region of North of France has the worst indicators in the country in terms of mortality [43] and quality of life [44].

### Strengths and limitations

One of the study’s strengths is that it used cross-sectional and longitudinal data from two large and nationally representative surveys, considering dozens of chronic and recurrent conditions and several health outcomes. Several limitations need also to be taken into account, including reliance on self-reported information on chronic or recurrent conditions, unmeasured or incompletely controlled confounding, and limited power to detect small effects associated with less frequent conditions or even moderate effects in longitudinal analyses. The use of self-reported information is especially subject to a number of biases, including a memory bias, an information bias associated with social desirability in the case of obesity and mental health conditions [45, 46] and a bias favoring more symptomatic conditions [47]. However, other recording methods such as medical sources (interviews or records) and especially administrative databases also have caveats, leading to low levels of agreements between self-reported, general practitioner-reported, and health administrative data among multimorbid patients [48, 49].

### Implication for public health policies

Efficiently reducing the burden of multimorbidity in the population requires a paradigm shift from predominantly disease-focused prevention efforts to integrated programs which consider the causes and mechanisms of aggregation of diseases and its timelines. The most common “elementary” aggregates of 2, 3 or 4 diseases (dyads, triads, tetrads, which concern 75% of multimorbid subjects in France [10]) need to be targeted early (no later than midlife) when multimorbid associations with health outcomes are the strongest.

**Fig 3:**
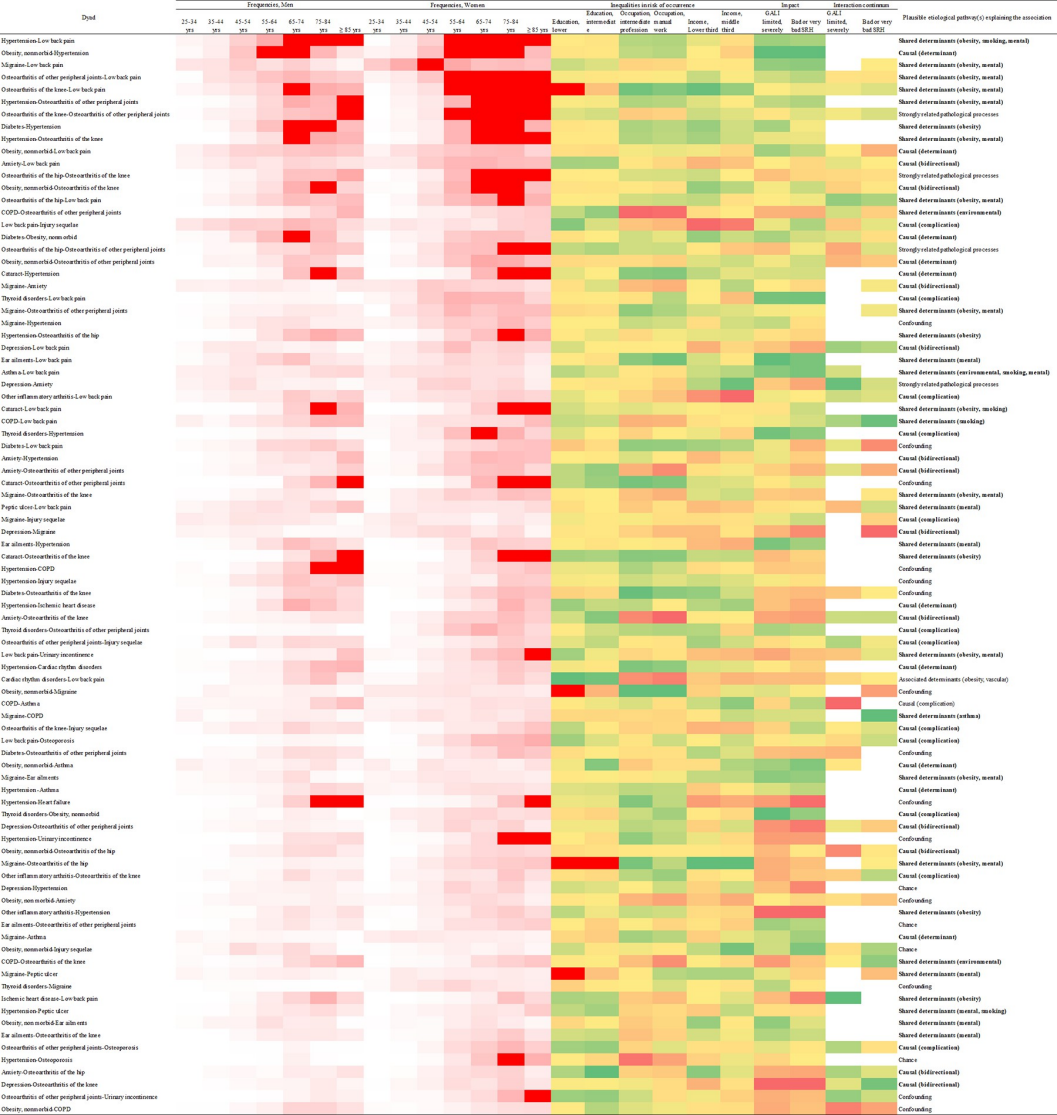
Summary characteristics of most frequent dyads (N = 88, ≥ 0.75% in HSM survey): Frequencies across age and gender categories (white to red gradient), inequalities in risk of occurrence (education, occupation, income), impact and interaction for GALI and SRH (green to red gradient), and plausible etiological pathway(s) explaining the association [see Coste et al. 2021] [10].

Fig 3 provides a graphical overview of the characteristics of the most frequent dyads, which can help prioritize targets for prevention. Of course, dyads with a greater impact on health status indicators or with strong multiplicative interaction, and those for which a leverage effect is possible (i.e., being causal for others or sharing risk factors) deserve special attention [10, 50]. Those which contribute early to the burden, at younger or middle age, and those marked by educational or occupational inequalities are also especially relevant.

Many of the most frequent dyads meet several of these criteria, especially the top-ranked ‘hypertension and low back pain’ (1^st^) and ‘nonmorbid obesity and hypertension’ (2^nd^)–both of which are driven by shared and causal determinants [51–55]–and the series of top-ranked associated painful conditions (migraine, low back pain and osteoarthritis of various joints) which share obesity and mental disorders as main determinants, and which may warrant concern in view of the likely increased risk of opioid dependency [56]. Top ranked dyads affected by socioeconomic inequalities, such as those including low back pain, osteoarthritis, chronic obstructive pulmonary disease and anxiety are also worth considering, especially given the causal relationship between education and health discussed above and the rich body of supporting literature [57–60]. Health education and promotion in their different aspects (physical and mental) and levels (population, individual) certainly offer valuable opportunities to counteract early multimorbidity development [61].

## Conclusions

Assessing and managing the burden of multimorbidity requires appropriate indicators, data sources, timelines, and level of analysis. Counting morbid entities in a closed list of conditions significantly impacting health status (with one or several categories “other”) seems unavoidable but is clearly insufficient. Our suggestion is to consider most frequent dyads (and triads) which are relevant in terms of impact, interaction and inequalities, as above detailed. The increasing availability of large healthcare databases represents an opportunity for multimorbidity assessment [62]. However, some key and highly multimorbid conditions such as obesity, musculoskeletal and mental disorders, may be unrecorded or unreliably recorded in some of these databases, and population surveys, targeting all adults will probably remain necessary, every 5 or 10 years, to capture many illnesses and sufferings. International, national and regional levels of analysis may all be relevant, but in every case, appropriate control for gender, age and socioeconomic status should be considered.

## Supporting information

S1 Table Frequencies of conditions involved in multimorbid associations across gender and age categories (HSM survey). (DOCX)

S2 Table Frequencies of dyads and triads involved in multimorbid associations across gender and age categories (HSM survey). (DOCX)

S3 Table Age and gender adjusted and fully adjusted (age, gender, education level, occupation, and household income) risk of multimorbidity (≥2 conditions), associated with urban unit and region as estimated in multiple binary logistic regression. (DOCX)

S1 Appendix STROBE checklist. (DOCX)
