# Supplementary material for: The epidemiology of multimorbidity in France: Variations by gender, age and socioeconomic factors, and implications for surveillance and prevention
Source: PLoS One. 2022 Apr 6;17(4):e0265842. doi: 10.1371/journal.pone.0265842 (PMC8986023; doi:10.1371/journal.pone.0265842)
Supplement: S1 Table — (DOCX) [file pone.0265842.s001.docx]

S1 Table. Frequencies of conditions involved in multimorbid associations across gender and age categories (HSM survey). All figures are weighted percentages.
