# Supplementary material for: The epidemiology of multimorbidity in France: Variations by gender, age and socioeconomic factors, and implications for surveillance and prevention
Source: PLoS One. 2022 Apr 6;17(4):e0265842. doi: 10.1371/journal.pone.0265842 (PMC8986023; doi:10.1371/journal.pone.0265842)
Supplement: S2 Table — (DOCX) [file pone.0265842.s002.docx]

S2 Table. Frequencies of dyads and triads involved in multimorbid associations across gender and age categories (HSM survey). All dyads and triads whose frequency is ≥ 0.50% are considered. Dyads and triads are presented in decreasing order of frequency. All figures are weighted percentages.

S2 Table (continued). Frequencies of dyads and triads involved in multimorbid associations across gender and age categories (HSM survey). All dyads and triads whose frequency is ≥ 0.50% are considered. Dyads and triads are presented in decreasing order of frequency. All figures are weighted percentages.

S2 Table (continued). Frequencies of dyads and triads involved in multimorbid associations across gender and age categories (HSM survey). All dyads and triads whose frequency is ≥ 0.50% are considered. Dyads and triads are presented in decreasing order of frequency. All figures are weighted percentages.

S2 Table (continued). Frequencies of dyads and triads involved in multimorbid associations across gender and age categories (HSM survey). All dyads and triads whose frequency is ≥ 0.50% are considered. Dyads and triads are presented in decreasing order of frequency. All figures are weighted percentages.
