# Supplementary material for: The epidemiology of multimorbidity in France: Variations by gender, age and socioeconomic factors, and implications for surveillance and prevention
Source: PLoS One. 2022 Apr 6;17(4):e0265842. doi: 10.1371/journal.pone.0265842 (PMC8986023; doi:10.1371/journal.pone.0265842)
Supplement: S3 Table — (DOCX) [file pone.0265842.s003.docx]

S3 Table. Age and gender adjusted and fully adjusted (age, gender, education level, occupation, and household income) risk of multimorbidity (≥2 conditions), associated with urban unit and region as estimated in multiple binary logistic regression. Odds ratios and 95% confidence intervals.
