# Supplementary material for: The epidemiology of multimorbidity in France: Variations by gender, age and socioeconomic factors, and implications for surveillance and prevention
Source: PLoS One. 2022 Apr 6;17(4):e0265842. doi: 10.1371/journal.pone.0265842 (PMC8986023; doi:10.1371/journal.pone.0265842)
Supplement: S1 Appendix — (DOCX) [file pone.0265842.s004.docx]

S4 Appendix. STROBE Checklist.

|  | | **Item No** | | **Recommendation** | | **Page No** | |
| --- | --- | --- | --- | --- | --- | --- | --- |
| **Title and abstract** | | 1 | | (*a*) Indicate the study’s design with a commonly used term in the title or the abstract | | Abstract, “Methods” section, **p. 2.** | |
|  |  |  |  | (*b*) Provide in the abstract an informative and balanced summary of what was done and what was found | | Abstract “Results” section, **p. 2.** | |
| **Introduction** | | | | | | | |
| Background/rationale | | 2 | | Explain the scientific background and rationale for the investigation being reported | | Introduction, beginning of the paragraph, **p. 3.** | |
| Objectives | | 3 | | State specific objectives, including any prespecified hypotheses | | Introduction, end of the paragraph, **p. 3.** | |
| **Methods** | | | | | | | |
| Study design | | 4 | | Present key elements of study design early in the paper | | Material and methods, “Survey designs, populations studied and collected data” section, beginning of the paragraph, **p. 3.** | |
| Setting | | 5 | | Describe the setting, locations, and relevant dates, including periods of recruitment, exposure, follow-up, and data collection | | Material and methods, “Survey designs, populations studied and collected data” section, beginning of the paragraph, **p. 3-4.** | |
| Participants | | 6 | | (*a*) *Cohort study*—Give the eligibility criteria, and the sources and methods of selection of participants. Describe methods of follow-up  *Case-control study*—Give the eligibility criteria, and the sources and methods of case ascertainment and control selection. Give the rationale for the choice of cases and controls  *Cross-sectional study*—Give the eligibility criteria, and the sources and methods of selection of participants | | Material and methods, “Survey designs, populations studied and collected data” section, middle of the paragraph, **p. 3-4.** | |
|  |  |  |  | (*b*) *Cohort study*—For matched studies, give matching criteria and number of exposed and unexposed  *Case-control study*—For matched studies, give matching criteria and the number of controls per case | | NA | |
| Variables | | 7 | | Clearly define all outcomes, exposures, predictors, potential confounders, and effect modifiers. Give diagnostic criteria, if applicable | | Material and methods, “Morbidity assessment” and “Health status measures” sections, **p. 4.** | |
| Data sources/ measurement | | 8* | | For each variable of interest, give sources of data and details of methods of assessment (measurement). Describe comparability of assessment methods if there is more than one group | | Material and methods, “Chronic and recurrent conditions” and “Outcomes” sections, **p. 4.** | |
| Bias | | 9 | | Describe any efforts to address potential sources of bias | | Material and methods, “Survey designs, populations studied and collected data” section, **p. 3-4.** | |
| Study size | | 10 | | Explain how the study size was arrived at | | Material and methods, “Survey designs, populations studied and collected data” section, **p. 3-54.** | |
| Quantitative variables | | 11 | | Explain how quantitative variables were handled in the analyses. If applicable, describe which groupings were chosen and why | | Material and methods, “Statistical analysis” section, second paragraph, **p. 6.** | |
| Statistical methods | | 12 | | (*a*) Describe all statistical methods, including those used to control for confounding | | Material and methods, “Statistical analysis” section, second paragraph, **p. 5.** | |
|  |  |  |  | (*b*) Describe any methods used to examine subgroups and interactions | | Material and methods, “Statistical analysis” section, second paragraph, **p. 5.** | |
|  |  |  |  | (*c*) Explain how missing data were addressed | | Material and methods, “Statistical analysis” section, second paragraph, **p.5.** | |
|  |  |  |  | (*d*) *Cohort study*—If applicable, explain how loss to follow-up was addressed  *Case-control study*—If applicable, explain how matching of cases and controls was addressed  *Cross-sectional study*—If applicable, describe analytical methods taking account of sampling strategy | | NA (Supplementary Table 1 indicates follow-up for 97% of the sample). | |
|  |  |  |  | (*e*) Describe any sensitivity analyses | | NA | |
| **Results** | | | | | | |  |
| Participants | 13* | | (a) Report numbers of individuals at each stage of study—eg numbers potentially eligible, examined for eligibility, confirmed eligible, included in the study, completing follow-up, and analysed | | “Survey designs, populations studied and collected data” section, **p. 3.** | |  |
|  |  |  | (b) Give reasons for non-participation at each stage | | “Survey designs, populations studied and collected data” section, **p. 3.** | |  |
|  |  |  | (c) Consider use of a flow diagram | | The flow diagram does not seem necessary in this particular case. | |  |
| Descriptive data | 14* | | (a) Give characteristics of study participants (eg demographic, clinical, social) and information on exposures and potential confounders | | Supplementary Table 1. | |  |
|  |  |  | (b) Indicate number of participants with missing data for each variable of interest | | Supplementary Table 1. | |  |
|  |  |  | (c) *Cohort study*—Summarise follow-up time (eg, average and total amount) | | Supplementary Table 1. | |  |
| Outcome data | 15* | | *Cohort study*—Report numbers of outcome events or summary measures over time | | Supplementary Table 1. | |  |
|  |  |  | *Case-control study—*Report numbers in each exposure category, or summary measures of exposure | |  | |  |
|  |  |  | *Cross-sectional study—*Report numbers of outcome events or summary measures | | Supplementary Table 1. | |  |
| Main results | 16 | | (*a*) Give unadjusted estimates and, if applicable, confounder-adjusted estimates and their precision (eg, 95% confidence interval). Make clear which confounders were adjusted for and why they were included | | Table 1 & 2, Supplementary Tables 3 & 6. | |  |
|  |  |  | (*b*) Report category boundaries when continuous variables were categorized | | Material and methods, “Outcomes” and “Statistical analysis” sections, **p. 4-5.** | |  |
|  |  |  | (*c*) If relevant, consider translating estimates of relative risk into absolute risk for a meaningful time period | | NA. | |  |
| Other analyses | 17 | | Report other analyses done—eg analyses of subgroups and interactions, and sensitivity analyses | | NA. | |  |
| **Discussion** | | | | | | |  |
| Key results | 18 | | Summarise key results with reference to study objectives | | Discussion, first paragraph, **p. 18.** | |  |
| Limitations | 19 | | Discuss limitations of the study, taking into account sources of potential bias or imprecision. Discuss both direction and magnitude of any potential bias | | Discussion, “Strengths and limitations” section, **p. 20.** | |  |
| Interpretation | 20 | | Give a cautious overall interpretation of results considering objectives, limitations, multiplicity of analyses, results from similar studies, and other relevant evidence | | Discussion, “Strengths and limitations” section, **p. 20.** | |  |
| Generalisability | 21 | | Discuss the generalisability (external validity) of the study results | | Discussion, “Discussion, “Implication for public health policies” section, **p. 20-1.** | |  |
| **Other information** | | | | | | |  |
| Funding | 22 | | Give the source of funding and the role of the funders for the present study and, if applicable, for the original study on which the present article is based | | Funding information was entered in the financial disclosure section of the submission system. | |  |
